# Beclin 1 and LC3 as predictive biomarkers for metastatic colorectal carcinoma

**DOI:** 10.18632/oncotarget.19939

**Published:** 2017-08-04

**Authors:** Hong Zhao, Maopeng Yang, Bin Zhao

**Affiliations:** ^1^ Harbin Medical University-Daqing, Heilongjiang, China; ^2^ Department of Medical Oncology, The Third Affiliated Hospital of Harbin Medical University, Heilongjiang, China; ^3^ The Second Affiliated Hospital and Yuying Children's Hospital, Wenzhou Medical University, Wenzhou, China

**Keywords:** colorectal carcinoma, beclin 1, LC3, metastasis, biomarker

## Abstract

Autophagy is a highly conserved self-destructive process that disassembles dysfunctional or unnecessary cellular components. It plays an important role in cancer metastasis, which is of particular interest considering metastatic disease is the major cause of colorectal carcinoma (CRC) related mortality. Here, we investigated the immunohistochemical expression of autophagy-related protein Beclin 1 and Microtubule-associated protein 1A/1B-light chain 3 (LC3) within surgical CRC specimens, first in a training cohort (205 patients), then in an inner validation cohort (160 patients) and an independent cohort (161 patients). The expressions of Beclin 1 and LC3 were lower in metastatic CRC compared with non-metastatic CRC. Furthermore, we developed an autophagy-based classifier for metastatic prediction. This classifier, including Beclin 1, LC3 and carcinoembryonic antigen (CEA) level, resulted in 82.9% sensitivity and 89.8% specificity for metastatic detection in the training cohort. In the independent cohort, it achieved 77.9% sensitivity and 90.3% specificity in predicting the metastasis of CRC. These results suggested that low expression of Beclin 1 and LC3 contributed to a more aggressive cancer cell phenotype, and our autophagy-based classifier was a reliable tool for metastatic prediction in CRC.

## INTRODUCTION

Colorectal carcinoma (CRC) is one common malignancies around the world [1]. Although most of the primary CRC can be removed by surgical resection, metastasis are often reported in distant organs such as the liver, lymph node, lung, peritoneum or bones [2]. It is reported that 25% of CRC patients have metastases at diagnosis and a further 33–50% develop metastases during the disease course [2]. In addition, the median survival in metastatic CRC is just approximately 24 months [3]. Currently, metastasis is generally believed as the leading cause of CRC related mortality, and early diagnosis of metastatic CRC is important for improving survivals. However, traditional clinic-pathologic markers for metastatic CRC, such as the depth of invasion [4], lymph node metastasis [5], venous invasion [6], CD44 [7], CD10 [8], matrix metalloproteinase 2 [9], transforming growth factor-α [9], vascular endothelial growth factor [9], and insulin-like growth factor II [9], only have limited predictive values.

Autophagy, an important intracellular homeostatic pathway for the degradation of dysfunctional organelles and proteins, can provide energy for survival under diverse cellular stresses [10, 11]. Despite accumulating evidence has demonstrated that autophagy plays a critical role in cancer metastasis, the underlying mechanisms is still unclear [12]. On the one hand, as an intracellular quality control system, autophagy can suppress pathological processes including cancer malignant translocation. On the other hand, it is also an adaptive strategy utilized by the tumor under various environment, suggesting its pro-metastatic role [13]. A better understanding the role of autophagy in metastatic CRC may unveil new predictive and prognostic biomarkers. Furthermore, identification of such markers will enable unique treatment of different subtypes of CRC and personalize patients’ management.

Multiple autophagy related genes and proteins are involved in metastatic CRC pathology [14, 15]. The Microtubule-associated protein 1A/1B-light chain 3 (LC3) family includes three isoforms (LC3A, LC3B and LC3C) [16]. LC3B accumulates specifically on nascent autophagosomes, and therefore, is one of the most reliable and widely used biomarker for autophagy [17]. In addition, LC3B was the first discovered autophagy related protein that involved in human CRC [18, 19]. Beclin 1, the mammalian orthologue of yeast Atg6, functions as a scaffold for the structure of phosphatidylinositol 3 kinase (PI3K) complex, which is the first step for autophagy process [20]. Numerous studies have shown that the expression levels of Beclin 1 are dysregulated in CRC tissues, especially in advanced stage tumor [19, 21, 22].

Our previous study has demonstrated the usefulness of an immunohistochemical score based on the autophagic proteins for survival prognosis in patients with CRC [19]. Here, we further examined the expression of autophagic protein Beclin 1 and LC3B in CRC tissue samples. Our results revealed that low expression of Beclin and LC3B, as determined by immunohistochemical analysis, associated with metastasis in CRC. Moreover, we developed an autophagy-based classifier for metastatic prediction. The main aim of this study is to explore the clinical significance of autophagy in cancer metastasis, therefore allowing more rational development of therapeutic strategies.

## RESULTS

### The correlation between Beclin 1/LC3B expression and metastatic CRC

**Figure 1:**
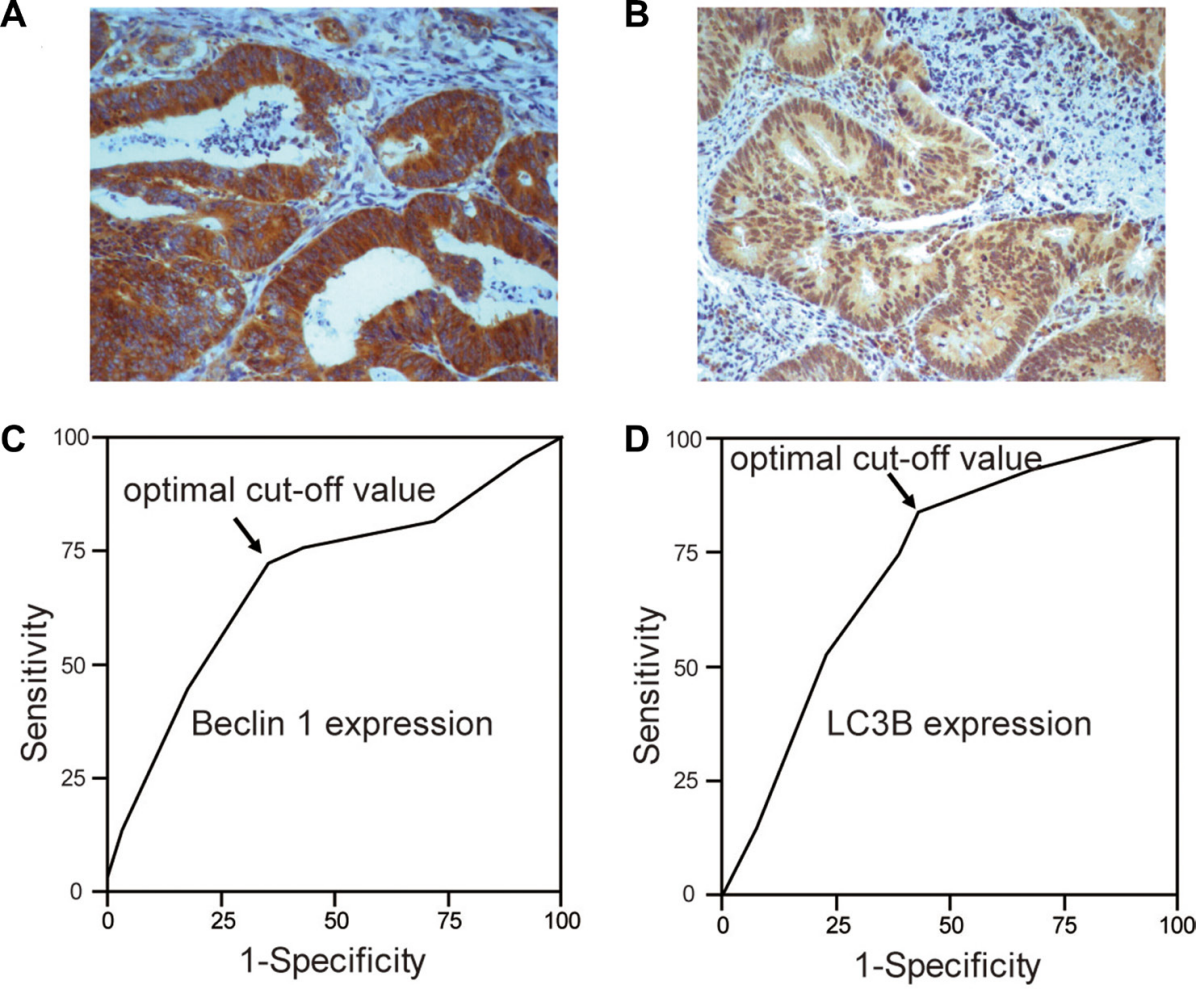
The expression of Beclin 1 and LC3B in CRC Examples of Beclin 1 (**A**) and LC3B (**B**) expression in CRC. Receiver operating characteristic (ROC) curves analysis of Beclin 1 (**C**) and LC3B (**D**) to determine the optimal cut-off points in the training cohort. At every immunohistochemical score, the 1-specificity and sensitivity for the metastasis were plotted to make the ROC curve. The cut-off scores for Beclin 1 and LC3B were 3.87 and 4.51, respectively.

A total of 526 patients with CRC were enrolled in this study, 226 of them had metastatic CRC. Immunohistochemical analysis was carried out to examine the protein expression of Beclin 1 and LC3B in all 526 tissues. As previously reported [19], both Beclin 1 and LC3B were expressed in most cancer tissues (Figure 1A and Figure 1B). Here, to further evaluate the predictive power of these two proteins in cancer metastasis, ROC curve analysis was conducted to determine the cut-off scores for Beclin 1 and LC3B in the training cohort.

For Beclin 1, the optimal cut-off score for metastasis in the training cohort (*n* = 205) was 3.87 (Figure 1C). Accordingly, we selected a Beclin 1 expression score of 4 (> 4 vs. < = 4) as the uniform cut-off point in the testing cohort and independent cohort. As shown in Table 1, low expression of Beclin 1 was mostly discovered in poorly differentiated colorectal cancer samples. Further correlation analysis revealed that the expression of Beclin 1 was associated with clinical stage, T stage, N stage, Carcinoembryonic antigen (CEA) level, carbohydrate antigen 19-9 (CA19-9) level in both the inner testing cohort and independent cohort. In addition, this study failed to detect any robust association between Beclin 1 and age, gender, disease subtype, and family history of cancer.

**Table 1:** Clinicopathological features of CRC subjects based on Beclin 1 expression in the training, testing and independent cohort

	Training cohort			Testing cohort			Independent cohort		
	High	Low	*P*	High	Low	*P*	High	Low	*p*
**No. of patients**	100	105		84	76		77	84	
**Age**			0.72			0.39			0.62
< 59	51	54		41	41		29	39	
> = 59	49	51		43	35		48	45	
**Gender**			0.12			0.37			0.39
Male	56	50		42	40		34	39	
Female	44	50		42	36		43	45	
**Disease type**			0.25			0.31			0.32
Colon	43	50		49	42		54	53	
rectal	57	55		35	34		23	31	
**Stage**			**< 0.01**			**< 0.01**			**< 0.01**
I + II	58	40		46	29		42	33	
III + IV	42	65		38	47		35	51	
**T stage**			**< 0.01**			**< 0.01**			**< 0.01**
T1 + T2	28	11		30	9		29	8	
T3 + T4	72	94		54	67		48	76	
**N stage**			**< 0.01**			**< 0.01**			**< 0.01**
N0 + N1	70	55		60	41		58	44	
N2 + N3 + Nx	30	50		24	35		19	40	
**Family history of cancer**			0.16			0.40			0.12
Yes	19	26		27	23		20	29	
No	81	79		57	53		57	55	
**Histology-differentiation**			**< 0.01**			**< 0.01**			**< 0.01**
Well	76	23		64	15		56	23	
Poorly	24	83		20	61		21	61	
**CEA (ng/ml)**			**< 0.01**			**< 0.01**			**< 0.01**
< = 5	75	48		59	35		55	34	
> 5	25	57		25	41		22	50	
**CA19-9(kU/L)**			**< 0.01**			**< 0.01**			**< 0.01**
< = 37	76	54		63	39		66	49	
> 37	24	51		21	37		11	35	
**Metastasis**			**< 0.01**			**< 0.01**			**< 0.01**
Yes	24	63		26	45		19	49	
No	76	42		58	31		58	35	

**Figure 2:**
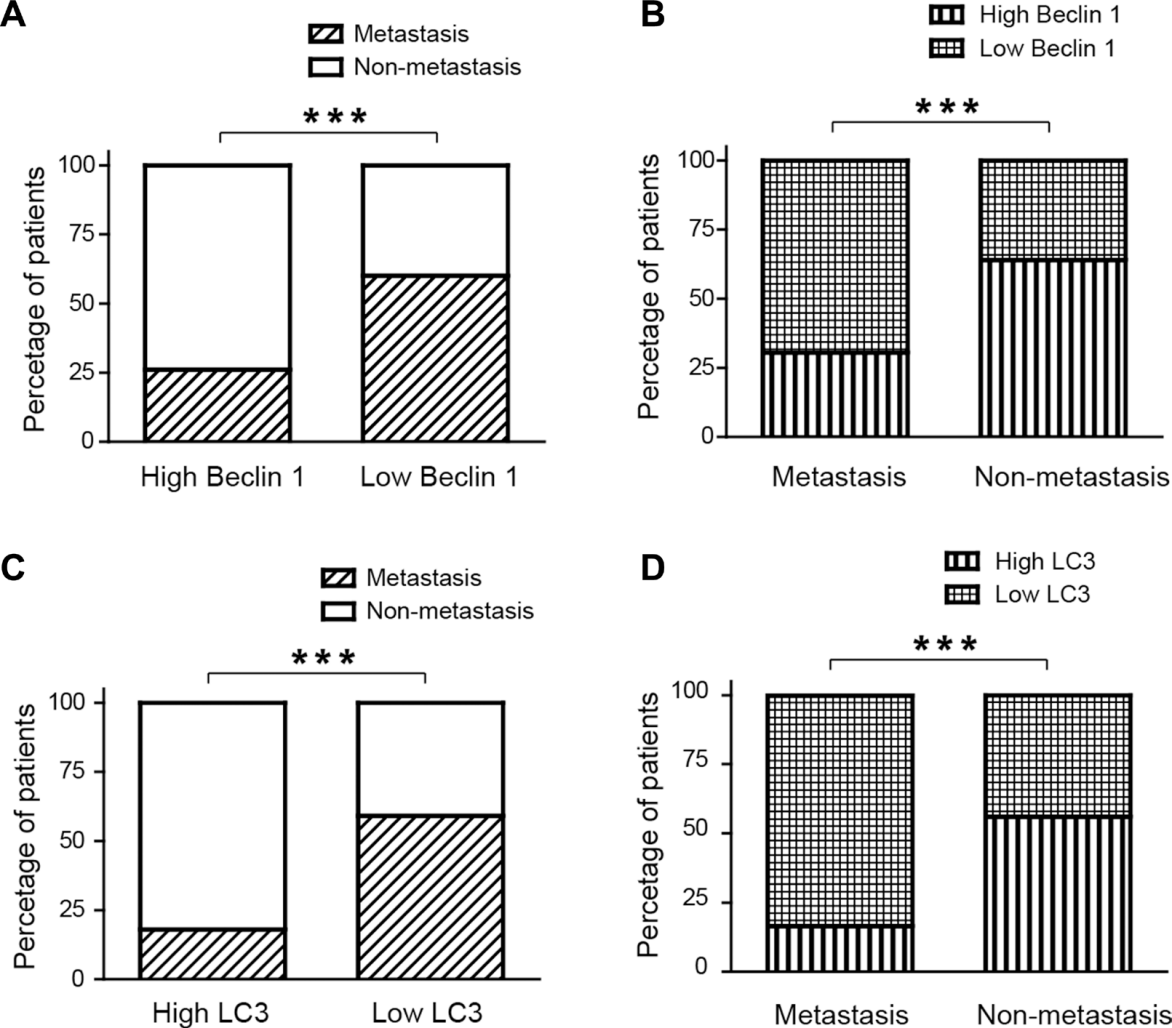
The expressions of Beclin 1 and LC3B were associated with metastatic colorectal cancer (**A**) In patients with Beclin 1 high expression, only 26% subjects had metastatic CRC. In contrast, 60% patients with Belin 1 low expression develop metastatic CRC. (**B**) The expressions of Beclin 1 in 74% metastatic CRC patients were low. On the contrary, 73% non-metastatic CRC patients highly expressed Beclin 1. (**C**) In patients with LC3B high expression, 18% subjects had metastatic CRC. In contrast, 60% patients with LC3B low expression develop metastatic CRC. (**D**) The expressions of Beclin 1 in 84% metastatic CRC patients were low. However, 56% non-metastatic CRC patients highly expressed Beclin 1.

Importantly, compared with patients with high expression of Beclin 1, subjects with low expression of Beclin 1 had more chance to develop metastatic CRC. (*p* < 0.001, Figure 2A). Consistent with this result, it is revealed that the expressions of Beclin 1 in 74% metastatic CRC patients were low. On the contrary, 73% non-metastatic CRC patients highly expressed Beclin 1 (*p* < 0.001, Figure 2B).

For LC3B, ROC analysis revealed that the optimal cut-off score was 4.51. Accordingly score value = 5 segregated the inner testing cohort as well as the independent cohort into low expression and high expression subgroup. As revealed in Table 2, the expression of LC3B was correlated with T stage, N stage, CEA level, CA19-9 level and histological differentiation. There was no robust correlation between LC3B expression and age, gender, disease subtype and family history of cancer.

**Table 2:** Clinicopathological features of CRC subjects based on LC3B expression in the training, testing and independent cohort

	Training cohort			Testing cohort			Independent cohort		
	High	Low	*P*	High	Low	*P*	High	Low	*p*
**No. of patients**	81	124		62	98		63	98	
**Age**			0.29			0.68			0.17
< 59	45	60		31	51		24	46	
> = 59	36	64		31	47		39	52	
**Gender**			0.07			0.40			0.31
Male	47	59		31	51		27	46	
Female	34	65		31	47		36	52	
**Disease type**			0.41			0.43			0.32
Colon	35	58		34	57		45	64	
rectal	46	66		28	41		18	34	
**Stage**			**< 0.01**			**< 0.01**			**< 0.01**
I+II	48	50		38	37		39	36	
III+IV	33	74		24	61		24	62	
**T stage**			**< 0.01**			**< 0.01**			**< 0.01**
T1 + T2	25	14		27	12		25	12	
T3 + T4	56	110		35	86		38	86	
**N stage**			**< 0.01**			**< 0.01**			**<0.01**
N0 + N1	58	67		51	50		52	50	
N2 + N3 + Nx	23	57		11	48		11	48	
**Family history of cancer**			0.11			0.32			0.34
Yes	14	31		18	32		18	31	
No	67	93		44	66		45	67	
**Histology-differentiation**			**< 0.01**			**< 0.01**			**< 0.01**
Well	57	42		46	33		48	31	
Poorly	24	82		16	65		15	67	
**CEA (ng/ml)**			**< 0.01**			**< 0.01**			**< 0.01**
< = 5	65	58		50	43		49	41	
> 5	16	66		12	55		14	57	
**CA19-9(kU/L)**			**< 0.01**			**< 0.01**			**< 0.01**
< = 37	67	63		55	47		56	59	
> 37	14	61		7	51		7	39	
**Metastasis**			**< 0.01**			**< 0.01**			**< 0.01**
Yes	14	73		11	60		12	56	
No	67	52		51	38		51	42	

Compared with patients with high expression of LC3B, subjects with low expression of LC3B had more chance to develop metastatic CRC (*p* < 0.001, Figure 2C). It was also demonstrated that the expression of LC3B in 84% metastatic CRC subjects were low, while 56% non-metastatic CRC patients highly expressed LC3B (*p* < 0.001, Figure 2D).

### Construction of classifier to predict the risk of metastasis of colorectal cancer

**Figure 3:**
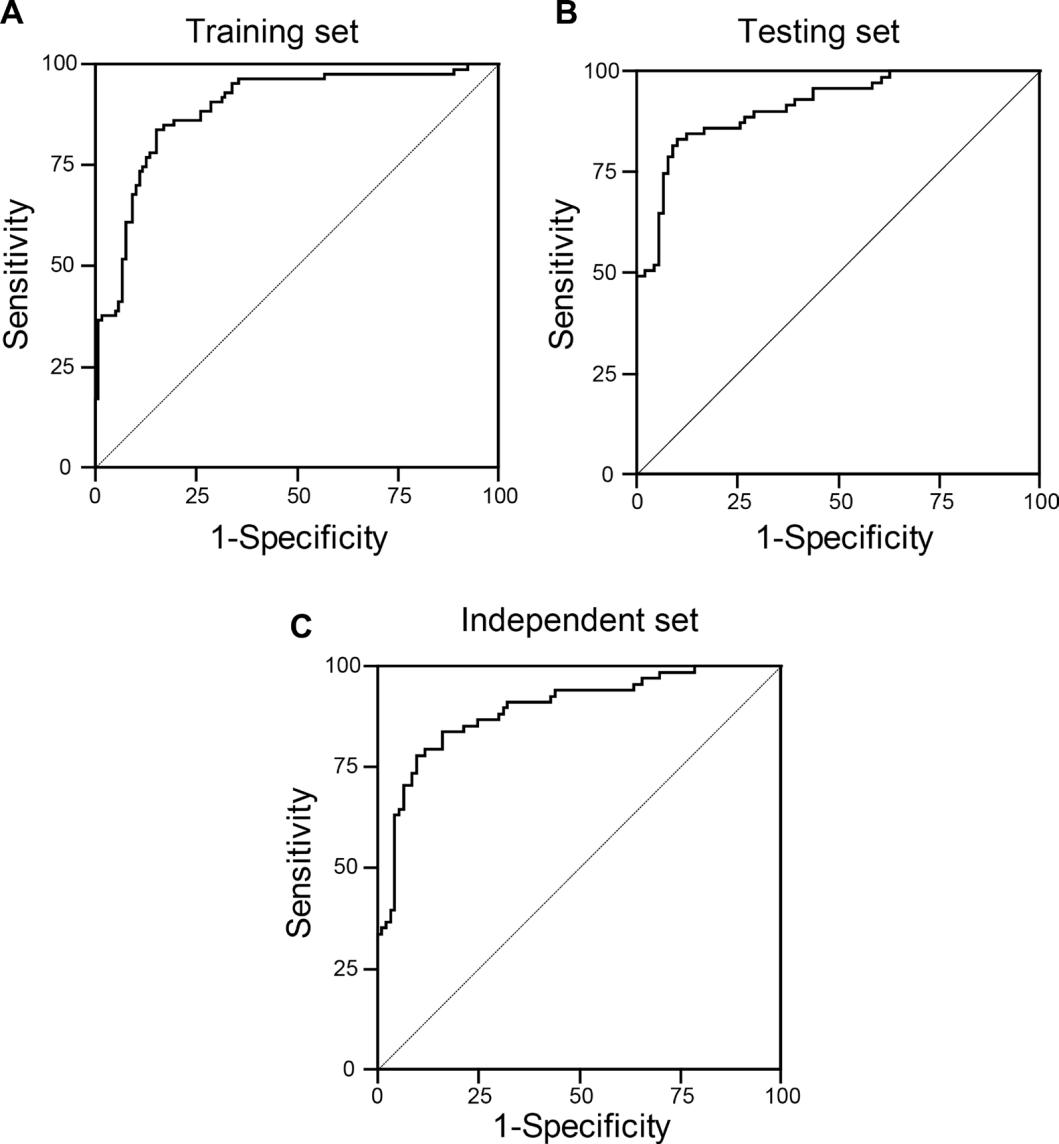
The receiver operating characteristics curve analysis of Beclin 1, LC3B in combination with serum CEA level in the training cohort (**A**), inner testing cohort (**B**), and independent cohort (**C**).

Logistic regression was carried out to investigate the significance of different clinic-pathological variables for metastasis prediction. It turned out that expression of Beclin 1 and LC3B, and the serum CEA level demonstrated predictive significance (*p* < 0.01) and increased the metastatic ratios (Table 3). To access the predictive biomarkers that achieved significance statistically in logistic regression analysis accurately, leave-one-out cross validation analysis was conducted. The results showed that the combination of Beclin 1, LC3B and CEA level yielded the most optimal specificity and sensitivity in the training set (Table 4). In addition, a classified equation was developed to measure the metastasis: Risk score = 0.585–0.269 × Beclin-0.377 × LC3B + 0.116 × CEA, “0” was defined as the cut-off point. This classified equation and cut-off point were then applied for the prediction in both the inner testing cohort and independent cohort (Figure 3). With Beclin 1 and LC3B expression, as well as the CEA level, this assay could predict the status of 184 cases in the training cohort correctly, with 21 cases were misclassified. The specificity and sensitivity were 89.8% and 82.9%, respectively (Table 4). We next calculated the ROC curve for the combination in both the inner testing cohort and independent cohort. As shown in Figure 3 and Table 4, the ability of Beclin 1, LC3B and CEA to classified metastasis CRC from non-metastasis CRC was statistically significant (*p* < 0.001), with the area under curve at 0.91 and 0.89, respectively.

**Table 3:** Multiple logistic regression analysis of different clinicopathological characteristics

	OR	95% CI	*P*
LC3	5.89	2.31–14.68	< 0.01
Beclin 1	4.43	1.82–10.29	< 0.01
CEA level	3.40	2.15–5.98	< 0.01
N stage	5.27	1.05–24.60	0.03
T stage	1.42	1.08–1.87	0.03
CA19-9	2.03	1.06–6.93	0.04

OR, odds ratio; CI, confidence interval.

**Table 4:** Receiver operating characteristic analysis of Beclin 1, LC3B in combination with serum CEA level

	AUC	95% CI	Sensitivity (%)	Specificity (%)
Training cohort	0.90	0.85–0.93	82.9	89.8
Inner testing cohort	0.91	0.86–0.94	82.1	89.9
Independent cohort	0.89	0.84–0.93	77.9	90.3

AUC, area under curve; CI, confidence interval.

## DISCUSSION

In this study, we analyzed the expression levels of autophagic protein Beclin 1 and LC3B, as determined by immunohistochemical labeling, in 526 CRC tissues. Our results showed that low expressions of Beclin 1 and LC3B were correlated with metastasis in colorectal cancer, suggesting Beclin 1 and LC3B contributed to the development of metastatic CRC. In addition, we developed an autophagy-based predictive model combining Beclin 1, LC3B and CEA level. The clinical application of this classifier could be a reliable index for metastatic prediction in CRC patients, and allowing more rational development of therapeutic strategies.

Metastasis is an important step in tumor progression, revealing the spread of malignant cancer cells from the original sites to distant organs and tissues [23]. The initial steps of metastasis such as invasion, colonization and intravasation often require some hallmarks at the primary tumor sites. Autophagy has an inhibitory effect during these early steps of metastasis through several suppressive mechanisms like restricting inflammation and necrosis, and establishing oncogene-induced senescence [24]. Inflammatory cells generate a favorable microenvironment for tumor metastasis during the development of cancer [25]. Necrosis can enhance the inflammation and promote inflammatory cell infiltration in response to hypoxia and metabolic stress [26]. By providing energy and metabolic precursors, autophagy effectively restricts necrosis and subsequent inflammatory cell infiltration, and thus inhibits cancer metastasis. In addition, the activation of oncogenic signals during cancer process can induce autophagy and it contributes to the establishment of oncogene-induced senescence [27]. Considering senescence is an important barrier to tumor development [28], this may be another process in which autophagy is required for tumor suppression.

The most extensively studied autophagy related genes/proteins in CRC are Beclin 1 and LC3 [15, 19, 29–33]. Ahn et al. first found the expression of Beclin 1 in 95% CRC samples [32]. However, no association between Beclin 1 expression and other clinicopathological characteristics (such as differentiation, histological type, location and gender) had been discovered [32]. In other studies, high expression of Beclin 1 had been correlated with better survival prognosis for patients with stage IIIB CRC treated with 5-fluorouracil based adjuvant chemotherapy [22]. In CRC patients with liver metastasis, it was reported that the expression of Beclin 1 had been inhibited [34]. Therefore Beclin 1 was considered as a biomarker for CRC metastasis [34]. The mechanism of Beclin 1 participate in CRC metastasis was still unclear. However, pre-clinical data showed that Beclin 1 could directly regulate tumor-associated inflammation through mediating immuno-modulatory factors like high-mobility group box protein1 (HMGB1), which was an extracellular signal in cancer metastasis [12]. HMGB1 played a crucial role in eliminating and neutralizing the reactive oxygen species (ROS) under oxidative stress [35]. When released, HMGB1 prevented metastasis, acting as an antitumor immune response.

Consist with previous studies [15, 19, 29–31], our results further disclosed the complicated interaction between autophagy and metastasis. Immunohistochemical analysis revealed that Beclin 1 and LC3B were highly expressed in the non-metastatic subgroup compared with metastatic subgroup. It was suggested that high expression levels of Beclin 1 and LC3B could inhibit the metastasis of CRC, Beclin 1 and LC3B hypo-expression might be employed to imply late development of CRC. These results were also confirmed from pre-clinical studies, which showed that Beclin 1 over-expression could reverse the aggressive phenotypes as a suppressor in CRC [34]. When examining the ability to predict colorectal cancer metastasis through logistic regression analysis, the expression of Beclin 1, LC3B and the serum CEA level were discovered to be accurate predictive biomarkers.

Traditional staging systems have been widely used to classify patients into different risk subgroup and to aid in cancer treatment. Although a number of CRC staging system (Astler-Coller classification, Dukes classification and TNM staging system) have been proposed, the efficacy of metastatic prediction still does not meet the clinical demand. As tumors are heavily heterogeneous, no individual gene seemed to be important for clinical significance and susceptibility to anti-tumor therapy. However, our proposed classifier, which integrates clinicopathological characteristics with autophagic proteins, revealed high predictive power for CRC patients who conduct resection. In addition, it provided a new strategy and approach for making optimal clinical decisions for patients with CRC. Clinicians could choose the optimal therapy that would maximize the therapeutic benefit. In addition, our data suggested that the expression of autophagic proteins, along with other clinicopathological characteristics, identified a clearer rationale for the function of autophagy in tumor metastasis.

The present study also had several limitations. First of all, this was a retrospective research; prospective studies involving long-term follow-up of CRC patients were needed to validate our results. Second, this research was conducted on Chinese patients only; the distribution of clinical characteristics might be different in other areas, making it susceptible to the inherent biases of such a study format. It would provide more information if other kinds of races are included. Third, only two major autophagic related proteins were analyzed in the present study because it is difficult to label all the proteins involved in autophagic processes. However, even with two proteins it is still possible to show significant prognostic efficacy that is supported by previous studies and biologically plausible.

In conclusion, our data showed that low expressions of Beclin 1 and LC3B were associated with the metastasis of CRC. Furthermore, we developed and validated a novel autophagy-based classifier for metastatic prediction. Prospective studies are required to evaluate this new marker for assessing the metastasis risk of CRC in the future work.

## MATERIALS AND METHODS

### Patients

We obtained 526 samples from surgery of pathologically proven colorectal carcinoma patients. For details of the study rationale, patient eligibility, clinical and histopathological features see our previous work [19]. For the training cohort set, 205 tissues were acquired from the Third Affiliated Hospital of Harbin Medical University. 160 other specimens obtain from the same hospital were included in the internal testing set. To further confirm our data, we also recruited 161 CRC patients from the Second Affiliated Hospital of Harbin Medical University as independent validation set. These patients included 261 males and 265 females, with a mean age of 59 years old (range, 28-92). Metastasis was detected by computed tomography (CT) scan, ultrasonography or observation at the time of surgery. All patients had follow-up records for over five years. Accordingly, we checked the metastatic status of these 526 patients five years after fist diagnosis in this study.

### Immunohistochemical analysis and evaluation

Immunohistochemical analysis was carried out to study the protein expression in colorectal cancer tissues. In brief, all formalin-fixed, paraffin-embedded (FFPE) tissues were carefully accessed by H&E staining to choose the most proper tumor sections. Then the selected sections were separated and deparaffinized in xylene and re-hydrated with ethanol solutions. The tissues were subsequently put in ethylene diamine tetra-acetic acid (EDTA) and autoclaved for five minutes. 3% H_2_O_2_ were applied to quench the endogenous peroxidase. Tissue sections were incubated at 4°C for overnight with a primary antibody for Beclin 1 (Abcam, UK), LC3B (Abcam, UK). The tissues were placed in peroxidase-conjugated streptavidin for 30 min before observe the final results with diaminobenzidine.

The immune-staining were accessed based on the method previously studied [6, 36]. In brief, the staining intensity was scored as follows: tissues without any staining were rated as 0, with faint staining as 1, with moderate staining as 2, and with strong staining as 3. The distribution of labeled protein was defined as the percentage accounting for the whole area and rated as follows: 0 (negative), 1 (1%–25%), 2 (26%–50%), 3 (51%–75%) and 4 (76%–100%). The total scores were determined by the combination of both distribution score and intensity score. The results of staining were independently evaluated by two researchers (H.Z. and M.Y.) who were blinded to the patients’ information.

To select the optimal cut-off scores of Beclin 1 and LC3B for metastasis, the receiver operating characteristic (ROC) curve analysis was carried out in the training cohort as previously reported [37]. Though minimizing the distance of the value to the top-left corner of ROC curve as well as maximizing the sum of sensitivity and specificity, the optimum cut-off value was determined. Based on the optimal cut-off values, Beclin 1 and LC3B level was investigated as a dichotomous variable for further evaluation.

### Statistical analysis

The associations between Beclin 1/LC3B expression and clinicopathological features were evaluated with two-sided t test, χ^2^ test, or Fisher's exact test as appropriate. To estimate the variables of clinicopathological features that may contribute to the prediction of metastasis, those with significant difference were evaluated by logistic regression analysis. Possible combinations of these factors were used to build up the optimal classifiers that could distinguish non-metastatic CRC from metastatic ones. The classifiers were determined by leave-one-out cross validation from patients in the training cohort. From this cross validation, the optimal value for a compound could be predicted, which in turn forecasted from the regression equation for all the other compounds. The discriminant formula and the cut-off value of the most satisfactory combination were then achieved to predict the probability of metastasis in the testing cohort and the independent cohort. ROC curves were performed to examine the predictive specificity, sensitivity and area under curve (AUC). All the analysis were carried out with IBM SPSS 20.0 and statistical significance was defined as *p* < 0.05 (two-tailed).
